# Supplementary material for: Mapping the molecular landscape of Lotus japonicus nodule organogenesis through spatiotemporal transcriptomics
Source: Nat Commun. 2024 Jul 29;15:6387. doi: 10.1038/s41467-024-50737-8 (PMC11289483; doi:10.1038/s41467-024-50737-8)
Supplement: Supplementary file 1 — Supplementary information [file 41467_2024_50737_MOESM1_ESM.pdf]

Supplementary Information

Mapping the Molecular Landscape of *Lotus japonicus* Nodule Organogenesis through Spatiotemporal Transcriptomics

Keyi Ye[1,10]*, Fengjiao Bu[1,10], Liyuan Zhong[2,10], Zhaonian Dong[1,10], Zhaoxu Ma[1,3,10], Zhanpeng Tang[1], Yu Zhang[1,4], Xueyong Yang[5], Xun Xu[6], Ertao Wang[7], William J. Lucas[1,8], Sanwen Huang[1,9], Huan Liu[2,6]*, Jianshu Zheng[1]*

[1]Shenzhen Branch, Guangdong Laboratory of Lingnan Modern Agriculture, Genome Analysis Laboratory of the Ministry of Agriculture and Rural Affairs, Agricultural Genomics Institute at Shenzhen, Chinese Academy of Agricultural Sciences, Shenzhen, Guangdong 518120, China.

[2]BGI Research, Wuhan 430074, China

[3]National Key Laboratory of Crop Genetic Improvement and National Centre of Plant Gene Research (Wuhan), College of Life Science and Technology, Huazhong Agricultural University, Wuhan 430070, China

[4]School of Agriculture, Sun Yat-sen University, Shenzhen 518107, China.

[5]State Key Laboratory of Vegetable Biobreeding, Institute of Vegetables and Flowers, Chinese Academy of Agricultural Sciences, Beijing 100081, China.

[6]State Key Laboratory of Agricultural Genomics, BGI Research, Shenzhen 518083, China.

[7]National Key Laboratory of Plant Molecular Genetics, CAS Center for Excellence in Molecular Plant Sciences, Institute of Plant Physiology and Ecology, SIBS, Chinese Academy of Sciences, Shanghai, China.

[8]Department of Plant Biology, College of Biological Sciences, University of California, Davis, CA 95616, USA.

[9]National Key Laboratory of Tropical Crop Breeding, Chinese Academy of Tropical Agricultural Sciences, Haikou, Hainan 571101, China

[10]These authors contribute equally to this work.
*Correspondence: yekeyi@caas.cn (K. Y.); liuhuan@genomics.cn (H. L.); zhengjianshu@caas.cn (J. Z.).

Supplementary Figures

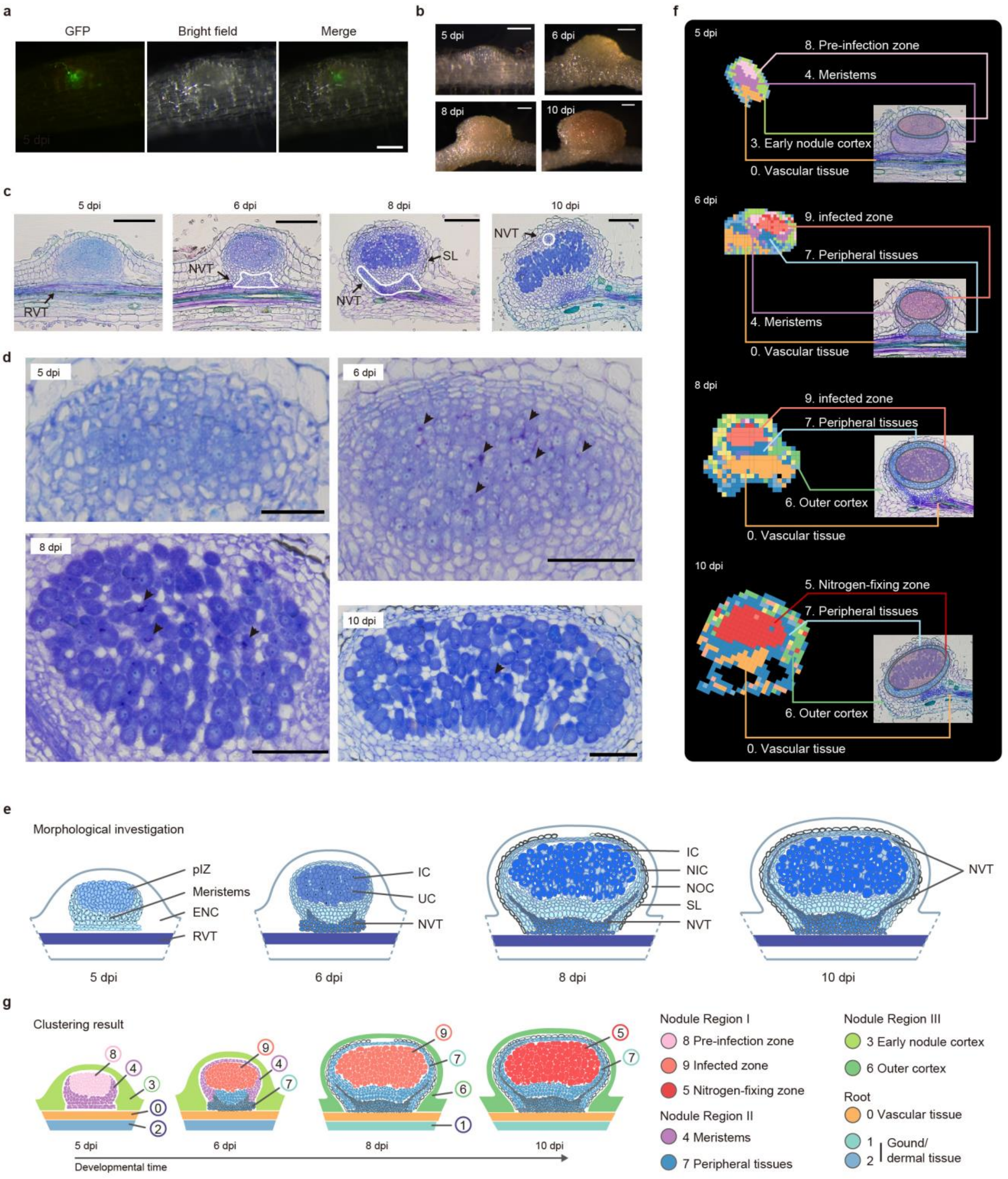

Supplementary Fig. 1 | Morphological and anatomical investigations on *L. japonicus* nodules.

(a) The fluorescence signal of GFP-labeled *M. loti* illustrating the harboring of the infection thread (IT) in the nodule primordium at 5 days-post-inoculation (dpi).

(b-c) Morphological and anatomical observations of nodules at different developmental stages. The image with representative events, at each stage, was chosen. Bright-field images are in (b) (scale bar = 100 μm); tissue slices with toluidine blue staining are shown in (c) (scale bar = 200 μm). The root vascular tissue is abbreviated as RVT; the scleroid layer is abbreviated as SL; the nodule vascular tissue (NVT) is indicated by white dashed lines.

(d) Representative images of IT branches at different developmental stages (scale bar = 100 μm). The IT branches are indicated by black arrowheads, and are most abundant at 6 dpi and barely detected at 10 dpi. The experiments in (a-d) were repeated three times with similar results.

(e) Sketch of the longitudinal sections of *L. japonicus* nodules illustrating the morphological and anatomical investigation and clustering results. Sketch of the morphological investigation is on the top. Abbreviations: pIZ (pre-infection zone), ENC (early nodule cortex), RVT (root vascular tissue), IC (infected cells), UC (uninfected cells), NVT (nodule vascular tissue), NIC (nodule inner cortex), NOC (nodule outer cortex), SL (scleroid layer). Based on (a-d), the development of nodule is as follows: By 5 dpi, the infection thread (IT) had already penetrated the root cortex (a) and induced protruding nodule primordia (b). The cells within this nodule meristems were tightly packed with large nuclei, exhibiting meristem characteristics (c). By 6 dpi, numerous IT branches were detected (d), and the darker staining reflected the appearance of infected cells (c). NVT, which interconnect the root and nodule, were detected at this stage (c). At 8 dpi, the nodule expanded (b), with increased bacteroids filling the infected cells; a scleroid layer formed separating inner cortex from outer cortex, and the NVT appeared enveloping the infection zone (c). Finally, at 10 dpi, the IT branches were barely detectable (d) and the nodule became fully functional, as indicated by its pink color (b).

(f) Detailed morphology-based annotation on main clusters. The spatial clustering images are linked with the tissue slice images, and key nodule tissues are circled.

(g) Sketch of the longitudinal sections of *L. japonicus* nodules illustrating the clustering result. Tissues are color-coded based on their assigned cluster and the tissue type of each cluster is listed on the right. The peripheral tissues mainly include nodule inner cortex and vascular tissues enveloping the infection zone. The early nodule cortex is the root cortical cells surrounding the nodule primordium, at the early stages, which will develop into the nodule outer cortex.

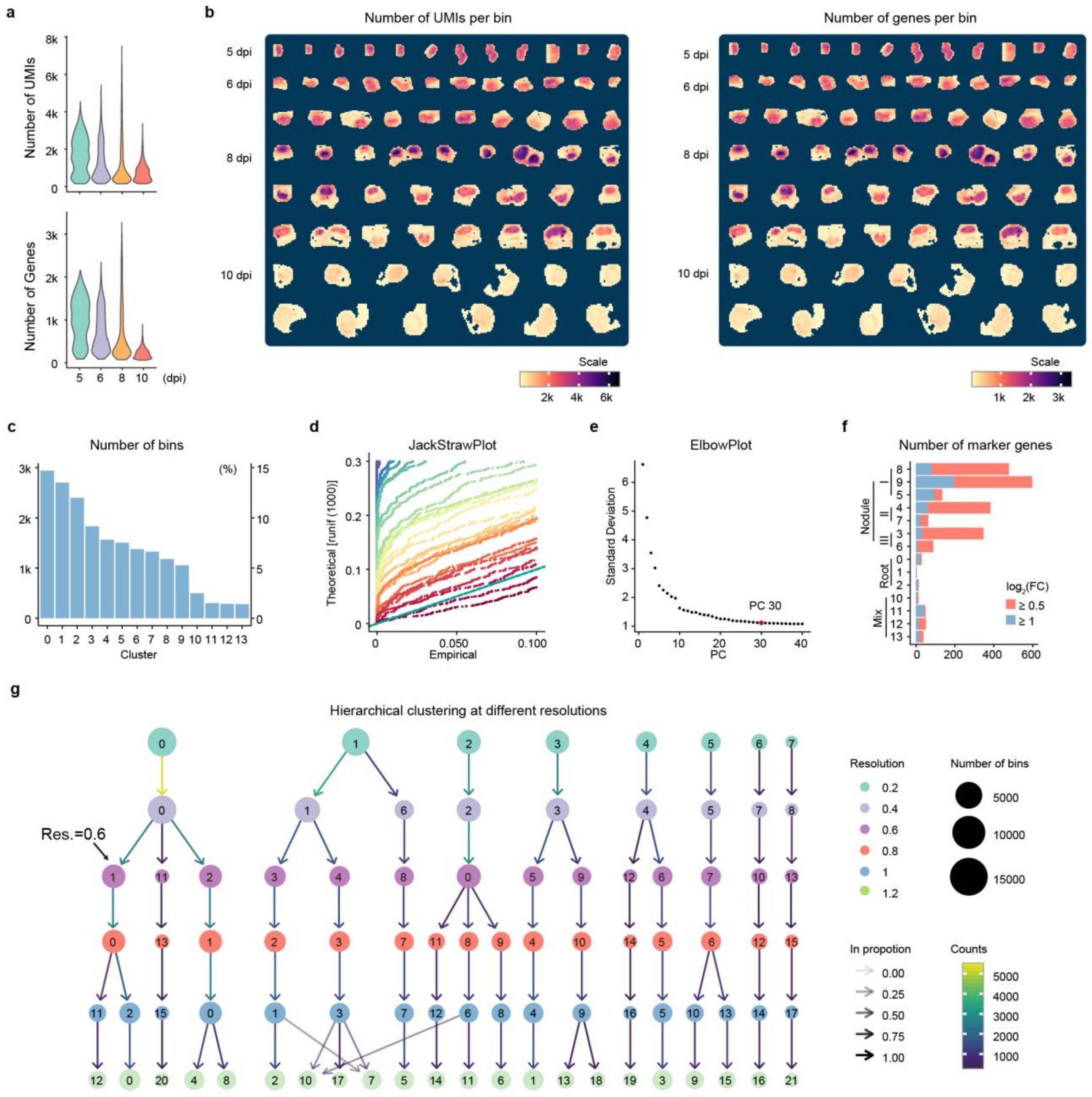

Supplementary Fig. 2 | Statistics of bins in spatial transcriptomes and selection of parameters in unsupervised clustering analysis.

(a) Violin plot illustrating the statistics for the number of UMIs and genes per bin.

(b) Spatial visualization of the number of UMIs and genes per bin.

(c) Bar plot showing the number and percentage of bins in each cluster.

(d) The plot illustrates the outcomes of the JackStraw analysis conducted to assess the significance of principal component analysis (PCA). In each quantile-quantile (QQ) plot, a specific subset of genes underwent random permutation, and subsequently, projected PCA scores were computed. These scores were then juxtaposed with the actual PCA scores to ascertain their statistical significance. The outcome revealed that principal components (PCs) 1 to 28 displayed significance with a P value of less than 0.01. Consequently, we selected the parameter, PC=30, for subsequent analysis within the Seurat package.

(e) The elbow plot aids in determining the appropriate number of principal components to retain for

further analysis within the Seurat package. The outcome clearly demonstrates that the explained variance after PC 30 is flat, indicating that utilizing 30 principal components sufficiently accounts for the variance.

(f) Bar plot showing the number of marker genes in each cluster. The cutoff of $\log_2(\text{Fold change}) \geq 0.5$ (pink) or ≥ 1 (cyan) are illustrated. The clusters are briefly annotated on the left. The full list of marker genes is provided in Supplementary Data 1

(g) Clustering trees showing hierarchical clustering at different resolutions in the unsupervised clustering analysis. The dendrogram obtained by clustering of bins at different resolutions, ranging from 0 to 1.2. Each bubble corresponds to a cluster. Different bubble colors represent the resolutions used for clustering, and the bubble size reflects the number of bins within this cluster. The color and Alpha of arrows, respectively, encode the number and percentage of bins assigned to clusters at the adjacent higher resolution. Finally, clustering result at resolution 0.6 (indicated by black arrow) was selected for subsequent investigation, which was based on agreement of morphological/anatomical observation with the clustering results.

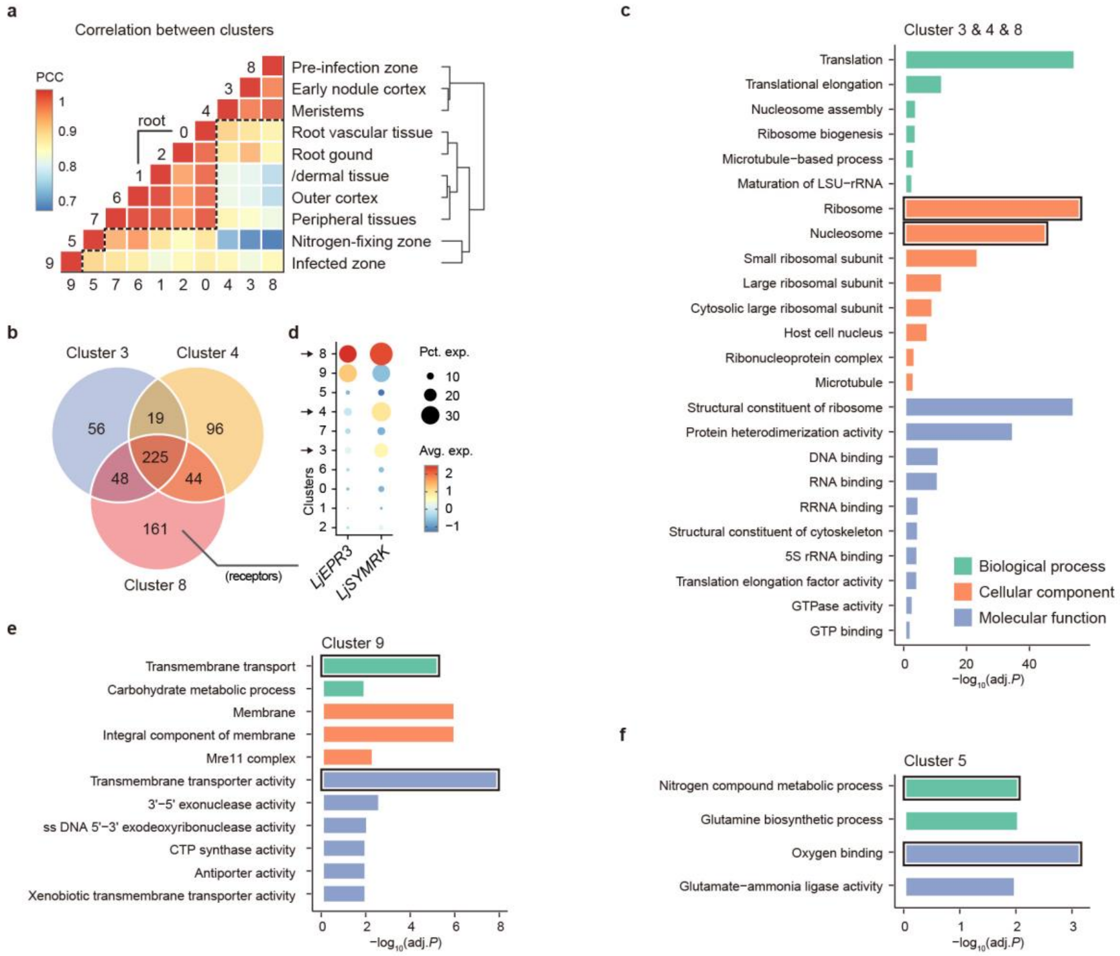

Supplementary Fig. 3 | Fulfilling symbiotic requirements results in distinction of the infection zone.

(a) Heatmap showing the Pearson correlation coefficient (PCC) between clusters 0 - 9. The color of squares encodes the PCC between indicated clusters. The clusters are primarily categorized into four modules delineated by dotted lines.

(b) Venn diagram showing the intersections of marker genes in clusters 3, 4 and 8. The numbers of genes in each set are labeled. The list of investigated genes that are distinctively expressed in each cluster is provided in Supplementary Data 2.

(c) Bar plot showing the representative GO enrichment results of 225 shared marker genes in clusters 3, 4 and 8. Significantly enriched GO terms are presented (adjusted P value < 0.05 ; relative enrichment > 1; counts > 1). The bubble size represents the counts of genes with indicated GO terms, and the color encodes $-\log_{10}$(adjusted P value). The detailed results of GO enrichment are shown in Supplementary Data 2.

(d) Bubble plot showing the clustering expression pattern of receptor genes distinctively expressed in cluster 8.

(e, f) Bar plots showing the GO enrichment results for the top 200 marker genes in cluster 9 (e) and 5 (f). The detailed results of GO enrichment are presented in Supplementary Data 2.

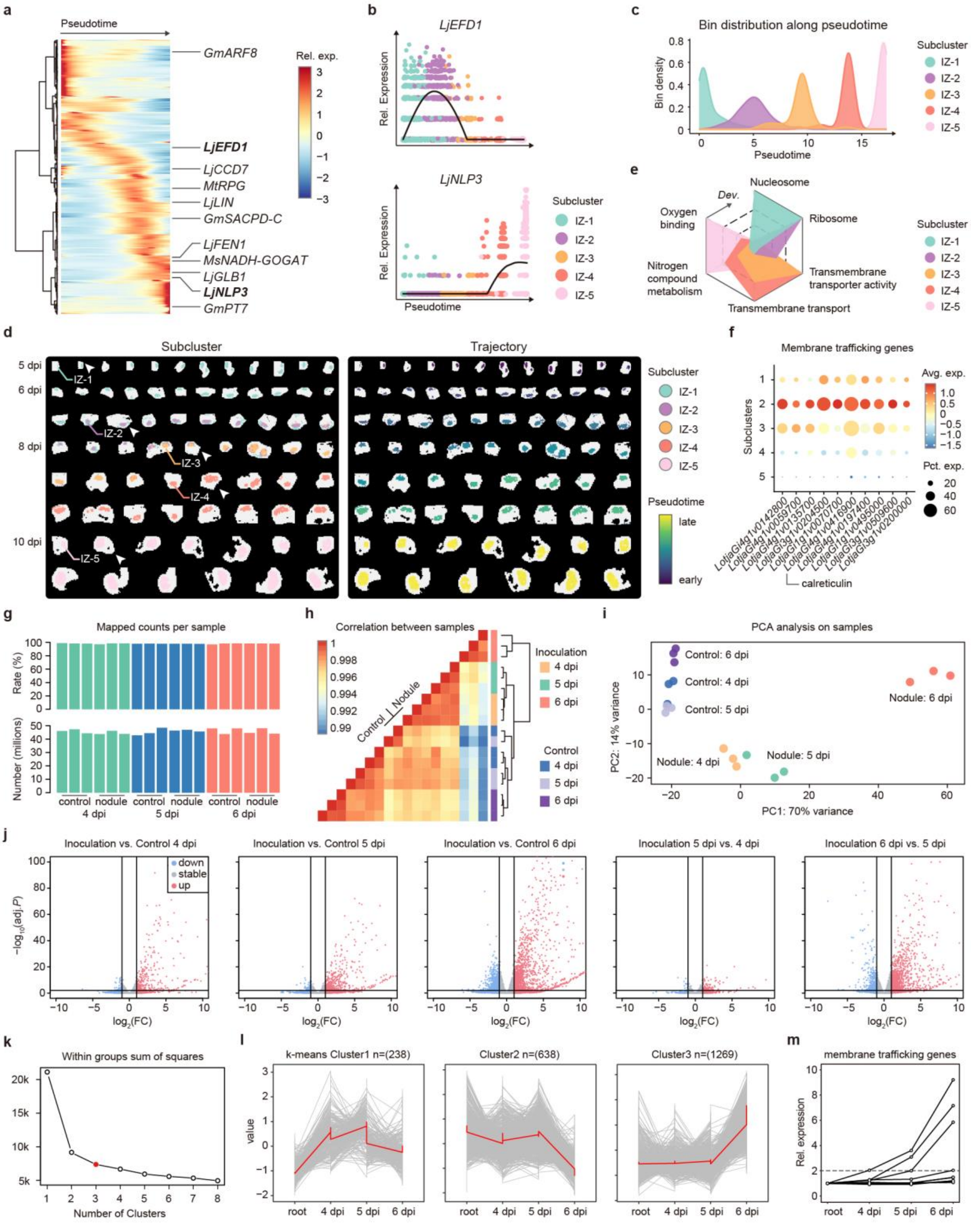

Supplementary Fig. 4 | Exploring infection zone development at the molecular level.

(a) The heatmap illustrating the dynamic changes in the expression levels of pseudotime-dependent genes across the pseudotime continuum. Genes investigated on nodulation, or their orthologs, are listed on the right. The full list is provided in Supplementary Data 3.

(b) The scatterplots depicting the pseudotemporal expression pattern of representative genes in (a). The smooth expression curves are represented by black curves.

(c) Area graph displaying the distribution of bins from each subcluster along pseudotime.
(d) Sub-clustering and spatial pseudotime trajectory results of bins in Fig. 2b (Monocle III). Samples indicated by white arrowheads are illustrated in Fig. 2f.
(e) Radar plot showing the relative enrichment of selected GO terms for the infection zone in each subcluster. The top 200 marker genes of each subcluster were used for the analysis. The enrichment score is relative to the highest one in each GO term and increases from inside to out in the plot. The arrowed black line indicates the developmental direction. The original data is provided in Supplementary Data 2.
(f) Bubble plot showing the clustering expression pattern of endocytosis related genes in the infection zone.
(g) Bar plots showing the number and ratio of reads mapped to Gifu genome in traditional transcriptomic analysis.
(h) Heatmap showing the Pearson correlation coefficient (PCC) between each sample in traditional transcriptomic analysis. The color of squares encodes the PCC between indicated clusters.
(i) PCA dimension reduction graph depicting the correlation between samples in traditional transcriptomic analysis.
(j) Volcano plot showing differentially expressed genes in traditional transcriptomic analysis. The cutoff was set as $\text{adjusted P value} < 0.05$ and $|\log_2(\text{FoldChange})| \geq 1$.
(k) The elbow plot indicating three clusters is appropriate for k-means clustering analysis in traditional transcriptomic analysis.
(l) Line graph showing the expression pattern of genes in each k-means cluster. The gray lines represent the gene expression pattern, and the red line indicates the expression tendency of all the genes.
(m) Line graph showing the expression pattern of endocytosis related genes in (f) determined by traditional transcriptomic analysis.

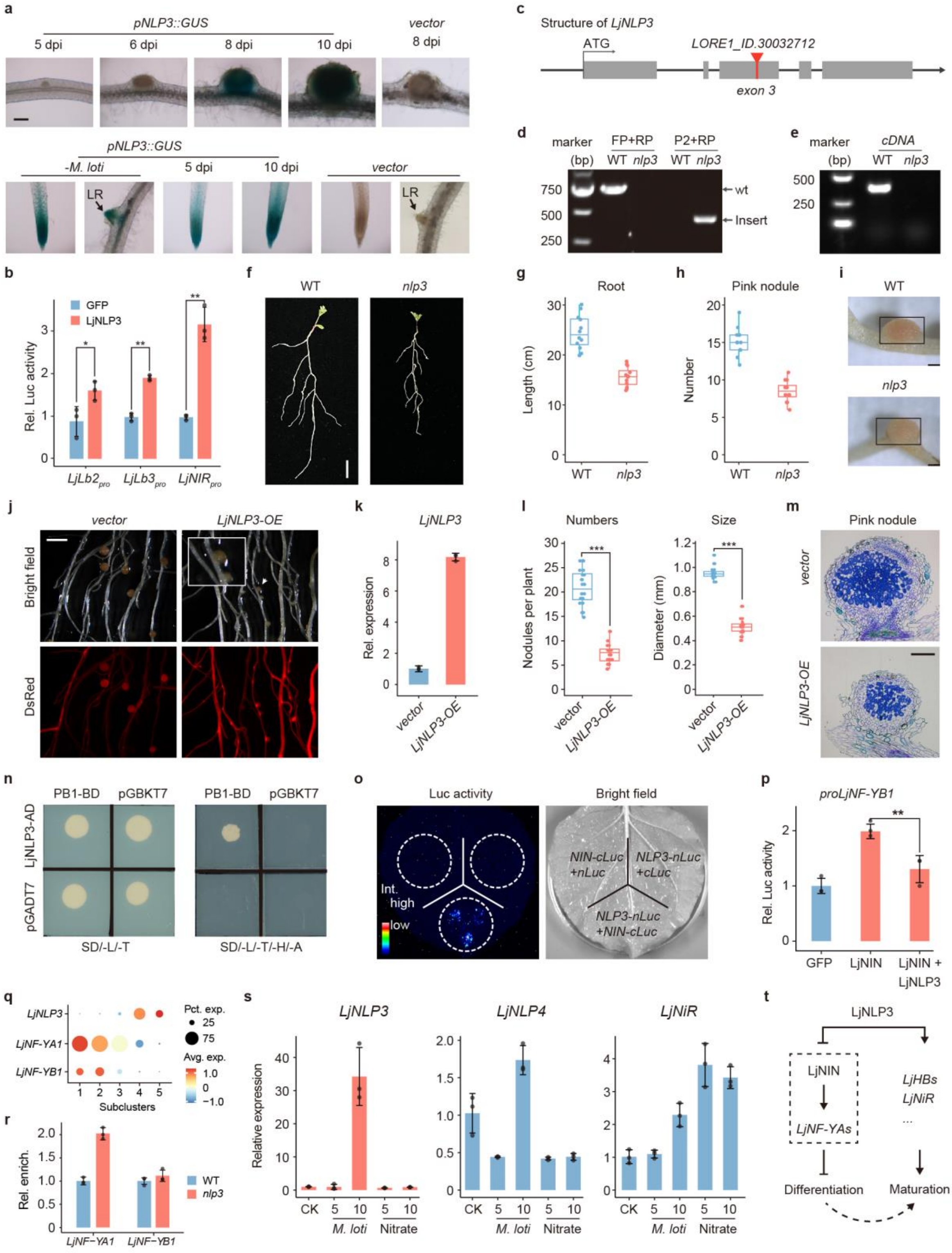

Supplementary Fig. 5 | LjNLP3 transitions nodules from the process of differentiation to maturation.

(a) GUS activity showing the expression pattern of *LjNLP3*. Hairy roots transformed with *pNLP3::GUS* were imaged, at the indicated dpi, after being inoculated with rhizobia. Hairy roots transformed with the vector were used as the control group (*vector*). Scale bar = 200 μm. All experiments presented in this section were replicated three times, yielding consistent results, and

source data (where applicable) are provided in the Source Data file.
(b) Dual-luciferase assays demonstrating that LjNLP3 induces the expression of leghemoglobins (*Lbs*). The investigation of *NIR* is also warranted since it has been reported to be induced by NLPs[1,2]. The GFP is employed as the control effector for LjNLP3. Data are normalized to samples expressing GFP, and represented as mean ± SD (n=3). **P value < 0.01; *P value < 0.05 (Student's *t* test).
(c) Gene structure sketch of *LjNLP3*. The red line denotes the *LORE1* insertion site in the *nlp3* mutant, identified with the plant ID 30032712.
(d) *LORE1* insertion identification in the *nlp3* mutant. Forward (FP), reverse primers (RP), and P2 primer were designed following the *Lotus* base protocol[3].
(e) Identification of *LjNLP3* transcripts in wild type (WT) and *nlp3* mutant plants. Primers for partial *exon 3* were designed to perform PCR, revealing the absence of *LjNLP3* transcripts in the *nlp3* mutant.
(f) Representative images of wild type (WT) and *nlp3* mutant plants at 10 dpi. Scale bar = 1 cm.
(g, h) Bar plots illustrating a decrease in root length (g) and number of pink nodules (h) in the *nlp3* mutant. Data are represented as mean ± SD (n=12).
(i) Representative images of wild type (WT) and *nlp3* mutant pink nodules at 10 dpi. Scale bar = 200 μm. According to previous studies and our investigation, mature nodules in *L. japonicus* typically exhibit a wider-than-taller morphology. The black squares indicate that pink nodules from WT are wider than those from *nlp3*.
(j) Representative images of hairy roots transformed with *35S::LjNLP3* (*LjNLP3-OE*), or empty vector (*vector*). Scale bar =2 mm. In the *LjNLP3-OE* group, the zoom-in image indicated by white arrowhead is presented for better visualization. The red inflorescence signal of DsRed roots, which is presented below, indicates successful transformation.
(k) The expression of *LjNLP3* in hairy roots transformed with *35S::LjNLP3*, or empty vector in (d). *LjUBQ* is used as the internal reference and data are represented as mean ± SD (n=3).
(l) Bar plots showing a significant reduction in nodule numbers and size in the *LjNLP3-OE* hairy roots in (d). The data are represented as mean ± SD (n=12). ***P value < 0.001 (Student's *t* test).
(m) Representative images of tissue-sectioned nodules in (d). The toluidine blue staining demonstrates that the infected cells in both groups are similar. Scale bar = 200 μm.
(n) Yeast two-hybrid assay revealing that LjNLP3 interacts with the PB1 domain of LjNIN.
(o) Luciferase complementation assays illustrating that LjNLP3 interacts with LjNIN. The left panel illustrates the intensity of luciferin (Int.); the right panel is the bright field image.
(p) Dual-luciferase assay showing that LjNLP3 inhibits LjNIN-induced expression of *LjNF-YB1*. Data are represented as mean ± SD (n=3). **P value < 0.01 (Student's *t* test).
(q) Bubble plot showing the clustering expression pattern of *LjNLP3* and *LjNF-Ys*. The result demonstrates that the expression of *LjNF-Ys* is depressed, when *LjNLP3* begins to be expressed.
(r) Real-time PCR indicating that *LjNF-Ys*' expression is elevated in the *nlp3* mutant. The wild type (WT) or *nlp3* mutant root materials, including nodules, at 10 dpi were sampled. The *LjUBQ* is used as the internal reference and data are represented as mean ± SD (n=3).
(s) Real-time PCR indicating that *LjNLP3* is specifically highly expressed in nodules at the late developmental stage. In the control group (CK), roots were collected without any treatment; in the *M. loti* groups, nodules inoculated with *M. loti* at 5 or 10 dpi, were sampled; in the Nitrate groups, roots, applied with 5 mM nitrate, were collected at 5 or 10 days-post treatment. The *LjUBQ* is used as the internal reference and data are represented as mean ± SD (n=3).
(t) A proposed model of the dual role played by LjNLP3 in nodule maturation process.

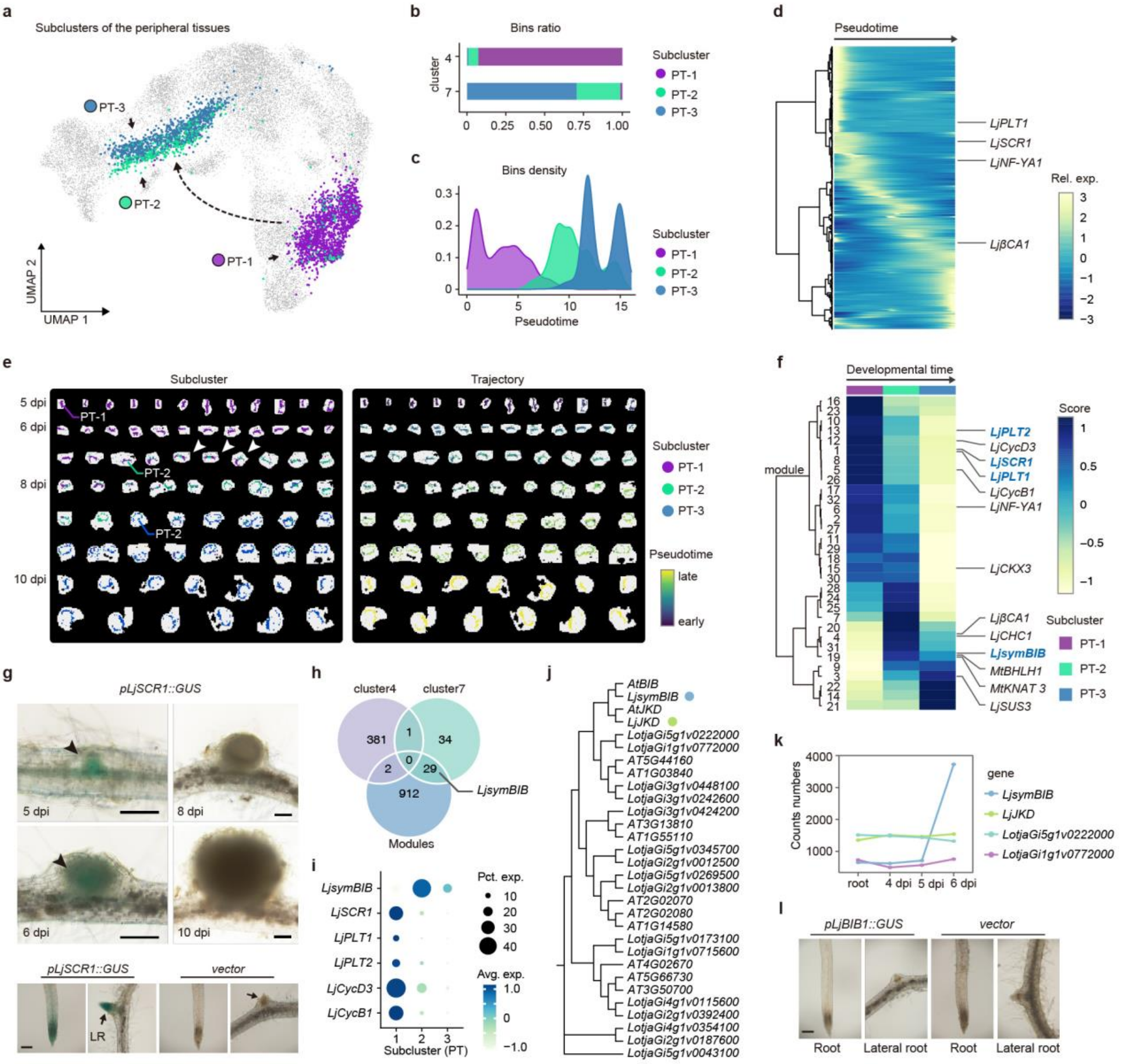

Supplementary Fig. 6 | SCR and PLT are promising candidates in regulating the progression of nodule meristems.

(a) The UMAP visualization of sub-clustering analysis on the meristems and peripheral tissues (clusters 4 and 7). The curve with an arrow indicates the developmental direction.

(b) Bar plot showing the proportion of subclusters within major clusters. The list of marker genes for each subcluster is provided in Supplementary Data 4.

(c) Area graph displaying the distribution of bins from each subcluster along pseudotime.

(d) The heatmap illustrating the dynamic changes in the expression levels of pseudotime-dependent genes across the pseudotime continuum. Genes investigated on nodulation, or their orthologs, are listed on the right. The full list is provided in Supplementary Data 4.

(e) Sub-clustering and spatial pseudotime trajectory results of bins in Fig. 3a (Monocle III). Bar plot showing the proportion of subclusters within major clusters 4 and 7. Samples indicated by white arrowhead demonstrated that peripheral tissue differentiation coincided with nodule vascular tissue emergence.

(f) Co-regulated modules of differentially expressed genes, based on the sub-clustering and pseudotime

trajectory results. The subclusters are arranged according to their developmental time. Genes investigated on nodulation, or their orthologs, are annotated on the right. The full list of these 4404 genes is available in Supplementary Data 4.
(g) GUS activity showing the expression of *LjSCR1*. Hairy roots transformed with *pLjSCR1::GUS* were imaged at the indicated dpi. The GUS signals in the nodule meristems are indicated by black arrowheads at 5 and 6 dpi. Scale bar = 200 μm. Experiments were repeated three times with similar results.
(h) Venn diagram showing the intersections of marker genes to clusters 4, 7 and genes from related co-regulated modules in (f) (modules 3, 4, 9, 19, 20 and 31).
(i) Bubble plot showing the clustering expression pattern candidate genes in the meristems and peripheral tissues.
(j) Phylogenetic tree of genes belonging to JKD family from *L. japonicus* and Arabidopsis.
(k) Line graph showing the expression of *LjsymBIB* and its paralogs.
(l) GUS activity showing that *LjsymBIB* is not expressed in root. Experiments were repeated three times with similar results.

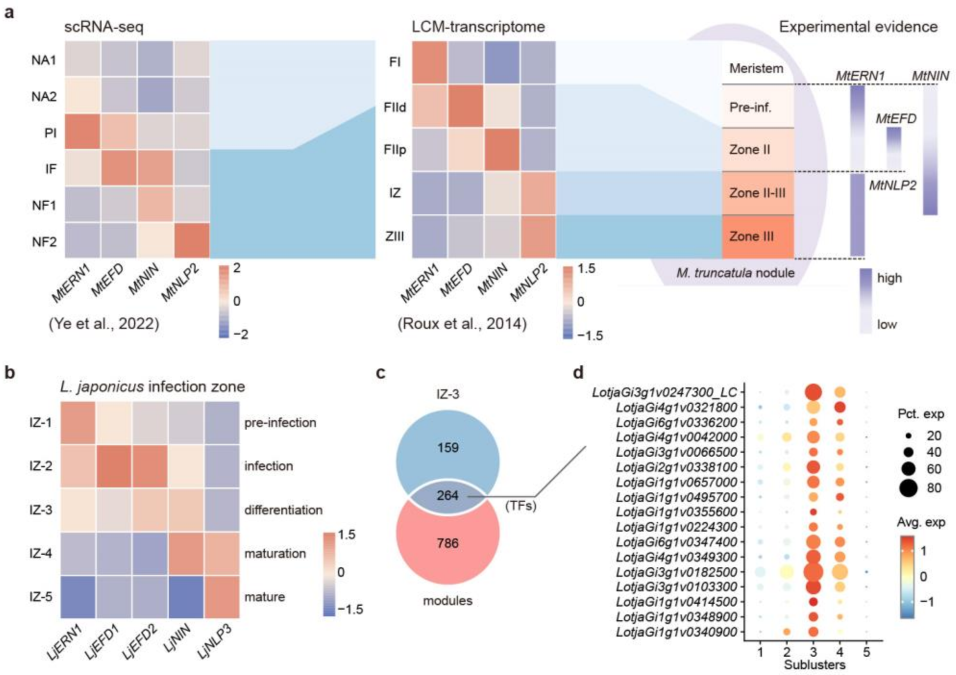

Supplementary Fig. 7 | Conserved regulatory genes imply evolutionary continuity and divergence between *L. japonicus* and *M. truncatula.*

(a) To comprehend the nodule developmental stages of *L. japonicus*, we drew upon studies conducted on *M. truncatula*. Based on previous research, we categorized the *M. truncatula* central region into 5 zones[4,5]. Several well-studied key TFs that can distinguish different zones in *M. truncatula*[2,4,6,7], with their orthologs from *L. japonicus* demonstrated in (a), indicated that the *M. truncatula* and *L. japonicus* share similar developmental trajectories.

(b) The expression pattern of candidate TFs in the infection zone of *L. japonicus* during nodule development.

(c) Venn diagram showing the intersections of marker genes in subclusters IZ-3 and genes from related co-regulated modules in Fig. 2e (modules 1, 7, 11, 23, 24, 26, 28).

(d) The expression pattern of candidate TFs regulating the IZ-3 stage.

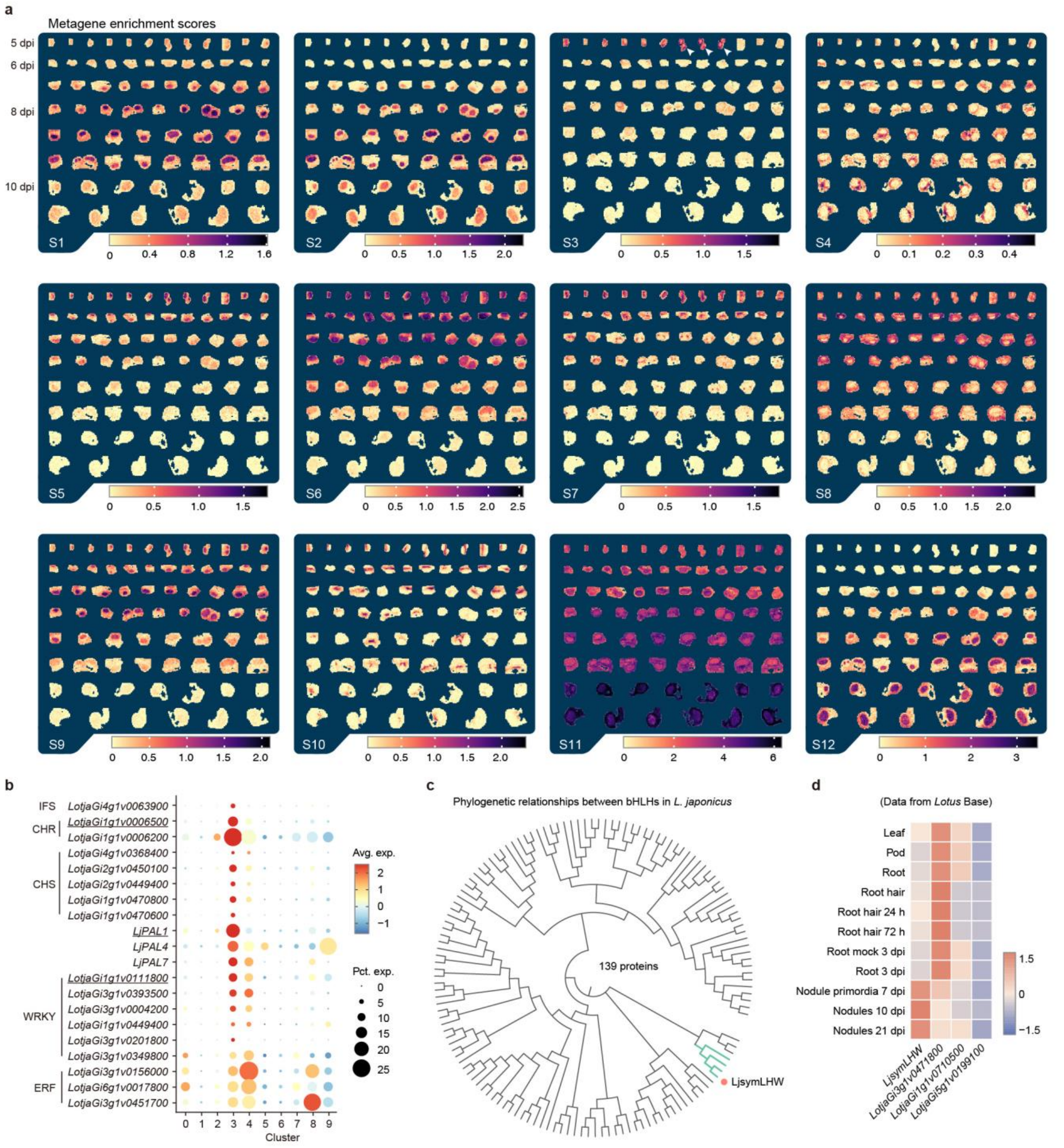

Supplementary Fig. 8 | Candidate genes revealed by spatial co-expression module analysis.

(a) The bin color encodes the metagene enrichment score, which can reflect the overall expression pattern of genes in the indicated modules. The module S3 shows an early expression pattern, as our samples are organized according to their developmental time and pseudotime. Samples indicated by white arrowheads in module S3 are illustrated in Fig. 4b.

(b) Bubble plot showing the clustering expression pattern of flavonoid-related genes and candidate regulators in module S3. The detailed information on these genes is presented in Supplementary Data 5.

(c) Phylogenetic tree of bHLH family in *L. japonicus*.
(d) Heatmap showing that *LjsymLHW* is mainly expressed in nodules. The data were retreated from *Lotus* Base[3].

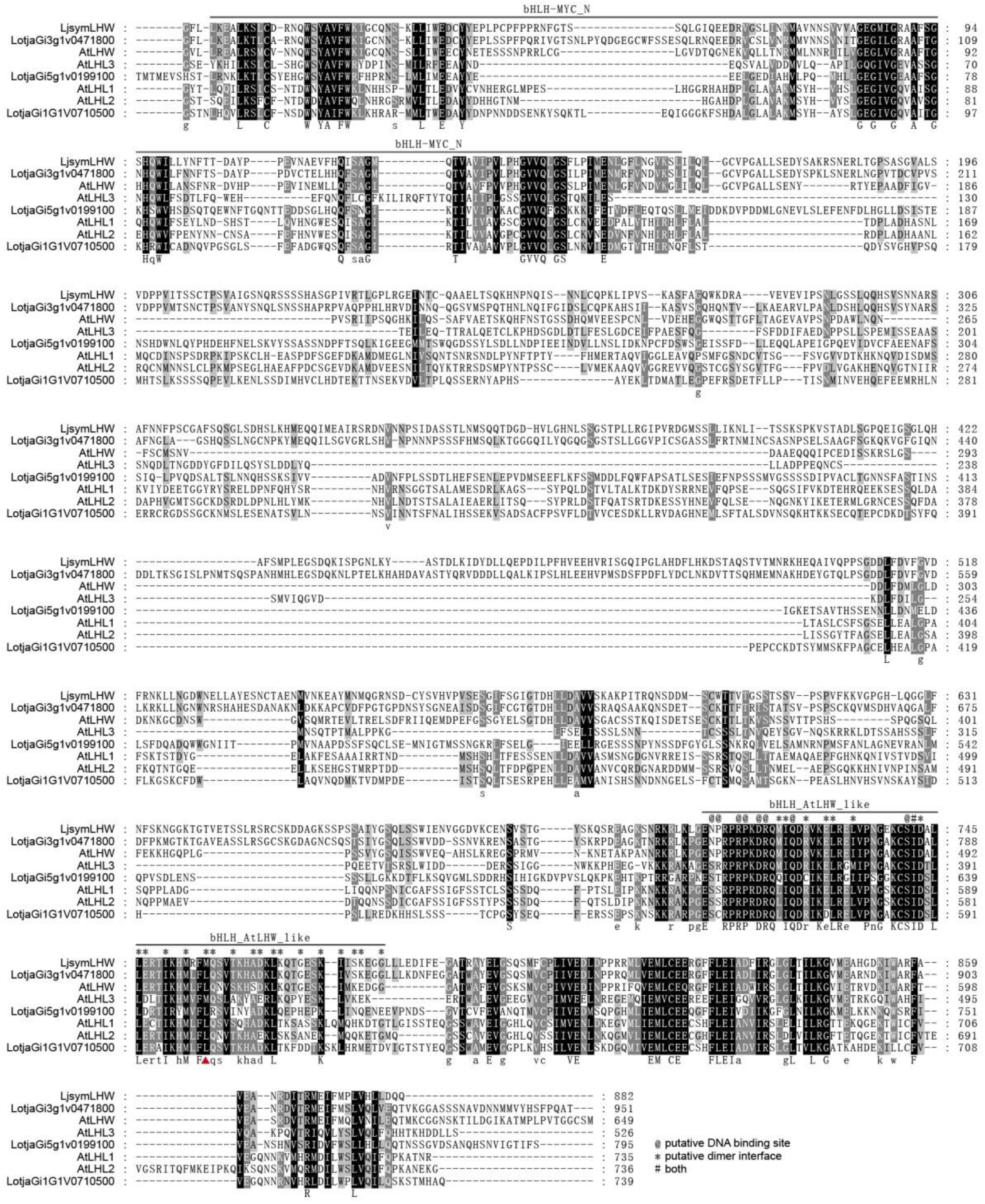

Supplementary Fig. 9 | Multiple sequence alignment of proteins belonging to LHW subfamily from *L. japonicus* and Arabidopsis.

The conserved domains, annotated on NCBI's Conserved Domain Database, are displayed at the top of the sequence, accompanied by markings of putative conserved amino acids. Notably, LjsymLHW features a rare amino acid at a putative dimer interface site, as indicated by the red triangle.

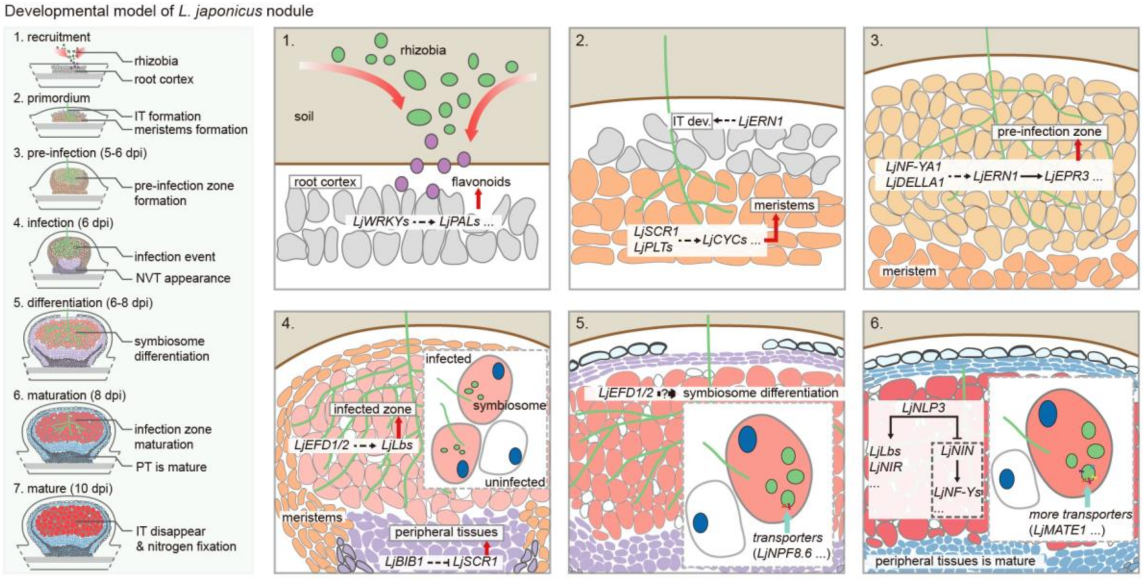

Supplementary Fig. 10 | Developmental model of *L. japonicus* nodules revealed by this study. At stage 1, LjWRKYs likely induce the expression of (iso)flavonoid enzymes, producing flavonoids to attract rhizobia. At stage 2, The formation of IT induces nodule meristem development, and LjSCR1 and LjPLTs likely regulate this process. At stage 3, pre-infection zone forms with LjERN1 participation. At stage 4, rhizobia infiltrate cells within the infection zone, leading to the emergence of symbiosomes. Concurrently, NVT commences its development from the root pericycle, while peripheral meristems proceed to develop, enclosing the central region. LjEFD1/2 and LjBIB1 regulate infection zone and peripheral tissue differentiation, respectively. At stage 5, peripheral meristems cease developing, leading to initial establishment of peripheral tissues. The expression of numerous transporters in the infection zone suggests symbiosome differentiation. At stage 6, peripheral tissues reach maturity, resulting in fully functional NVT, while infection zone enters maturation process under LjNLP3 regulation. Finally, at stage 7, infection zone achieves full maturity and nodule initiates nitrogen fixation.

Supplementary References

1 Soyano, T., Shimoda, Y. & Hayashi, M. NODULE INCEPTION antagonistically regulates gene expression with nitrate in Lotus japonicus. *Plant Cell Physiol.* **56**, 368-376, doi:10.1093/pcp/pcu168 (2015).

2 Jiang, S. *et al.* NIN-like protein transcription factors regulate leghemoglobin genes in legume nodules. *Science* **374**, 625-628, doi:10.1126/science.abg5945 (2021).

3 Mun, T., Bachmann, A., Gupta, V., Stougaard, J. & Andersen, S. U. Lotus Base: An integrated information portal for the model legume *Lotus japonicus*. *Sci. Rep.* **6**, 39447, doi:10.1038/srep39447 (2016).

4 Ye, Q. *et al.* Differentiation trajectories and biofunctions of symbiotic and un-symbiotic fate cells in root nodules of *Medicago truncatula*. *Mol. Plant* **15**, 1852-1867, doi:10.1016/j.molp.2022.10.019 (2022).

5 Roux, B. *et al.* An integrated analysis of plant and bacterial gene expression in symbiotic root nodules using laser-capture microdissection coupled to RNA sequencing. *Plant J.* **77**, 817-837, doi:10.1111/tpj.12442 (2014).

6 Liu, J. Y. *et al.* NIN is essential for development of symbiosomes, suppression of defence and premature senescence in *Medicago truncatula* nodules. *New Phytol.* **230**, 290-303, doi:10.1111/nph.17215 (2021).

7 Vernie, T. *et al.* EFD Is an ERF transcription factor involved in the control of nodule number and differentiation in *Medicago truncatula*. *Plant Cell* **20**, 2696-2713, doi:10.1105/tpc.108.059857 (2008).
