# Supplementary material for: Mapping the molecular landscape of Lotus japonicus nodule organogenesis through spatiotemporal transcriptomics
Source: Nat Commun. 2024 Jul 29;15:6387. doi: 10.1038/s41467-024-50737-8 (PMC11289483; doi:10.1038/s41467-024-50737-8)
Supplement: Supplementary file 3 — Description of Additional Supplementary Files [file 41467_2024_50737_MOESM3_ESM.pdf]

Description of Additional Supplementary Files

Supplementary Data Legends:

Supplementary Data 1. Information on the spatial transcriptomic data and lists of marker genes for major clusters, related to Fig. 1 and Supplementary Fig. 2. Each sheet contains quality assessment information, complete marker genes list, or annotations for investigated genes.

Supplementary Data 2. Original data of GO enrichment analyses, related to Supplementary Fig. 3 and 4e. Each sheet contains original data of GO enrichment analysis.

Supplementary Data 3. Lists of marker genes and co-regulated genes of the infection zone, related to Fig. 2 and Supplementary Fig. 4. Each sheet contains complete marker genes list, co-regulated genes list, or annotations for investigated genes.

Supplementary Data 4. Lists of Marker genes and co-regulated genes of the meristems and peripheral tissues, related to Fig. 3 and Supplementary Fig. 6. Each sheet contains complete marker genes list, co-regulated genes list, or annotations for investigated genes.

Supplementary Data 5. Lists of genes in each spatial co-expression module, related to Figure 4 and Supplementary Fig. 8. Each sheet contains complete spatial co-expression gene list, or annotations for investigated genes.

Supplementary Data 6. Information on vectors and primers used in this article, related to Methods section.
